# Risk Factors for Spotted Fever Group Rickettsioses in Kilimanjaro Region, Tanzania

**DOI:** 10.1093/ofid/ofae664

**Published:** 2024-11-13

**Authors:** Thomas R Bowhay, Matthew P Rubach, Ângelo J F Mendes, William L Nicholson, Jamie L Perniciaro, Michael J Maze, Ganga S Moorthy, Jo E B Halliday, Kathryn J Allan, Blandina T Mmbaga, Wilbrod Saganda, Bingileki F Lwezaula, Rudovick R Kazwala, Sarah Cleaveland, Katrina J Sharples, Venance P Maro, John A Crump

**Affiliations:** Centre for International Health, University of Otago, Dunedin, New Zealand; Division of Infectious Diseases and International Health, Department of Medicine, Duke University Medical Center, Durham, North Carolina, USA; Duke Global Health Institute, Duke University, Durham, North Carolina, USA; Programme in Emerging Infectious Diseases, Duke–National University of Singapore, Singapore; Department of Medicine, Kilimanjaro Christian Medical Centre, Moshi, Tanzania; School of Biodiversity, One Health and Veterinary Medicine, College of Medical Veterinary and Life Sciences, University of Glasgow, Glasgow, United Kingdom; Rickettsial Zoonoses Branch, Division of Vector-Borne Diseases, National Center for Emerging and Zoonotic Infectious Diseases, Centers for Disease Control and Prevention, Atlanta, Georgia, USA; Rickettsial Zoonoses Branch, Division of Vector-Borne Diseases, National Center for Emerging and Zoonotic Infectious Diseases, Centers for Disease Control and Prevention, Atlanta, Georgia, USA; Department of Medicine, University of Otago, Christchurch, New Zealand; Duke Global Health Institute, Duke University, Durham, North Carolina, USA; Division of Infectious Diseases, Department of Pediatrics, Duke University Medical Center, Durham, North Carolina, USA; School of Biodiversity, One Health and Veterinary Medicine, College of Medical Veterinary and Life Sciences, University of Glasgow, Glasgow, United Kingdom; School of Biodiversity, One Health and Veterinary Medicine, College of Medical Veterinary and Life Sciences, University of Glasgow, Glasgow, United Kingdom; Department of Medicine, Kilimanjaro Christian Medical Centre, Moshi, Tanzania; Department of Paediatrics and Child Health, Kilimanjaro Christian Medical University College, Tumaini University, Moshi, Tanzania; Department of Medicine, Mawenzi Regional Referral Hospital, Moshi, Tanzania; Department of Medicine, Mawenzi Regional Referral Hospital, Moshi, Tanzania; Department of Veterinary Medicine and Public Health, Sokoine University of Agriculture, Morogoro, Tanzania; School of Biodiversity, One Health and Veterinary Medicine, College of Medical Veterinary and Life Sciences, University of Glasgow, Glasgow, United Kingdom; Department of Mathematics and Statistics, Division of Sciences, University of Otago, Dunedin, New Zealand; Department of Medicine, Dunedin School of Medicine, Division of Health Sciences, University of Otago, Dunedin, New Zealand; Department of Medicine, Kilimanjaro Christian Medical Centre, Moshi, Tanzania; Department of Paediatrics and Child Health, Kilimanjaro Christian Medical University College, Tumaini University, Moshi, Tanzania; Centre for International Health, University of Otago, Dunedin, New Zealand; Division of Infectious Diseases and International Health, Department of Medicine, Duke University Medical Center, Durham, North Carolina, USA; Duke Global Health Institute, Duke University, Durham, North Carolina, USA; Department of Paediatrics and Child Health, Kilimanjaro Christian Medical University College, Tumaini University, Moshi, Tanzania

## Abstract

**Background:**

Knowledge gaps exist on risk factors for spotted fever group rickettsioses (SFGR) in sub-Saharan Africa. We sought to identify SFGR risk factors in Kilimanjaro Region, Tanzania.

**Methods:**

We recruited febrile patients presenting at 2 hospitals in Moshi from February 2012 through May 2014. Standardized clinical and risk factor questionnaires were administered. SFGR exposure was defined as a *Rickettsia africae* immunofluorescence antibody reciprocal titer ≥64, and acute SFGR as a ≥4-fold rise between paired sera. Logistic regression was used to identify associations.

**Results:**

Of 1190 participants providing ≥1 serum sample, the median age was 21.8 (range, 0.3–100.2) years, 646 (54.3%) were female, and 650 (54.6%) had SFGR exposure. Of 731 participants with paired sera, 67 (9.2%) had acute SFGR. On multivariable analysis, odds of acute SFGR were higher in the age group 0–2 years (adjusted odds ratios [aORs] for older age groups, <0.36; *P* < .011), rural residence (aOR, 4.1; *P* = .007), and areas with maximum daily temperature <26°C (aORs for higher temperature groups, <0.42; *P* < .035). Odds of SFGR exposure were higher in those working in the garden (aOR, 1.8; *P* = .010) and seeing a dog (aOR, 1.5; *P* = .010). Odds of SFGR exposure were lower in the age group 0–2 years (aORs for older age groups, >1.5; *P* < .026), female sex (aOR, 0.62; *P* < .001), and being from the Chaga tribe (aOR, 0.68; *P* = .003).

**Conclusions:**

Those aged <2 years, rural residents, and persons residing in areas with cooler temperatures had increased odds of SFGR. Our results identify groups for further research on tick exposure and for targeted prevention interventions.

Rickettsial diseases are a common cause of severe febrile illness in northern Tanzania, where spotted fever group rickettsioses (SFGR) predominate [1, 2] and annual SFGR incidence exceeds 100 per 100 000 persons [3]. To design effective prevention and control measures, an understanding of risk factors for infection is needed, yet there has been little published research on SFGR risk in sub-Saharan African countries. Research among travelers returning from sub-Saharan Africa has identified an association with game hunting and visiting rural areas [4–6]. Studies done in African countries have focused on seroprevelance of SFGR or SFGR exposure rather than acute infections, identifying increased odds of exposure among males, those residing rurally, and persons working in agriculture [7–9].

Both *Rickettsia conorii*, the agent of Mediterranean spotted fever, and *Rickettsia africae*, the agent of African tick bite fever, are present in Tanzania [10, 11]. Both present with fever, rash, headache, myalgia, regional lymphadenopathy, and variable presence of eschar. Mediterranean spotted fever is associated with a number of severe complications that are less common in African tick typhus [5, 12]. *Rickettsia conorii* is principally transmitted by the brown dog tick *Rhipicephalus sanguineus*, which feeds on dogs, whereas *R africae* is transmitted by the tropical bont tick *Amblyomma variegatum*, which feeds on ruminant livestock [13]. There may be undiscovered SFGR in Tanzania with distinct ecologies. Contact with cattle has been found to be associated with increased odds of exposure to SFGR in South Africa and Tanzania [9, 14, 15]. The role of climate and geography as determinants of SFGR risk is poorly understood, but these factors likely mediate risk through modifying tick distribution and behavior. Temperature is known to influence tick survival, host preference, and changes in questing behavior [16–19]. Higher land temperature has been linked to increased SFGR seroprevalence in southern Tanzania [9].

We sought to identify risk factors for both acute SFGR and SFGR exposure among patients presenting to hospital in the Kilimanjaro Region of Tanzania. Our goal was to identify factors on the causal pathway that could be amenable to targeted prevention interventions.

## MATERIALS AND METHODS

### Setting

We undertook a prospective observational hospital-based surveillance study of febrile illness at Kilimanjaro Christian Medical Centre (KCMC), a 450-bed zonal referral hospital, and Mawenzi Regional Referral Hospital (MRRH), a 300-bed regional referral hospital, in Moshi, Tanzania. At the time of the study, the population of Moshi municipality was 184 000 and the regional population was 1.6 million [20]. Moshi sits at approximately 890 meters above mean sea level and has a tropical wet and dry climate, with 2 wet seasons typically occurring from October through December and from March through May. The Kilimanjaro Region has a diverse range of habitats, including cultivated flatlands surrounding Moshi, areas of shrubland, and mountains with montane forest. Aside from the Moshi urban area, the Kilimanjaro Region is predominantly rural [20], with agriculture and livestock husbandry occurring broadly in pastoral, smallholder, or agropastoral systems [21].

### Study Participants and Procedures

The methods for study population sampling have been presented in detail elsewhere [22, 23]. In brief, potential participants were identified among pediatric and adult patients presenting to KCMC and MRRH from February 2012 through May 2014. From Monday through Friday and within 24 hours of presentation, we screened all patients at the adult medical ward at KCMC and the adult and pediatric medical wards and outpatient department at MRRH. Inpatients and outpatients were eligible for enrollment if they had a tympanic temperature of ≥38°C, and inpatients reporting a fever in the 72 hours prior to presentation were also eligible. After obtaining written informed consent, a standardized clinical history and risk factor questionnaire was administered by a study team member (Supplementary Appendix 1). The risk factor questionnaire included data on sociodemographic characteristics and environmental exposures, including to dogs and livestock. For participants who reported a place of residence in the Kilimanjaro Region, study personnel visited participant households to record the Global Positioning System (GPS) coordinates. When GPS data could not be obtained at the household level, the GPS coordinate centroid of the participant's reported village or neighborhood of residence was used. Blood was collected for serum at enrollment and participants were asked to return 4–6 weeks after enrollment for collection of a convalescent serum sample.

### Laboratory Methods

Serum specimens were stored at −70°C and were batch shipped on dry ice from Moshi, Tanzania, to Atlanta, Georgia, United States. At the Rickettsial Zoonoses Branch of the US Centers for Disease Control and Prevention, indirect immunofluorescence antibody (IFA) testing was performed using *R africae* antigen on all available samples. Starting at 1:32, an end-point titer, defined as the highest titer displaying specific fluorescence, was obtained via serial 2-fold dilution. IFA provides information on the infecting rickettsial group but not the infecting rickettsial species within the group [24].

### Case Definitions

Acute SFGR was defined as a participant with a ≥4-fold rise in *R africae* IFA immunoglobulin G (IgG) titer between acute and convalescent serum [25]. SFGR exposure was defined as a participant with a serum IFA IgG reciprocal titer of ≥64 in either acute or convalescent serum [2].

### Geospatial and Climate Data

The participant locations were assigned a population density in persons per square kilometer (km^2^) using data from WorldPop [26]. Urban areas were defined as those with ≥1000 persons/km^2^, periurban areas were defined as >300 to <1000 persons/km^2^ and located within 20 km of urban areas, and all others were classified as rural [27]. Similarly, based on participant household location, mean monthly rainfall for the calendar year of hospital admission, mean monthly rainfall of the 3 months prior to hospital admission, mean monthly minimum and maximum temperature of the year of hospital admission, and the mean monthly minimum and maximum temperature of the 3 months prior to hospital admission were extracted from WorldClim [28]. Mean normalized difference vegetation index (NDVI) for the year of hospital admission and the mean NDVI of the 3 months prior to admission were obtained from the Terra Moderate Resolution Imaging Spectroradiometer indices [29], and elevation was obtained from Shuttle Radar Topography Mission data [30]. Cattle, goat, and sheep density in head per km² were extracted from the Gridded Livestock of the World (GLW 2) database [31].

### Statistical Methods

The analysis was restricted to residents of the Kilimanjaro Region. To inform variable selection and model building, we visualized causal assumptions [32]. A socioeconomic status score was derived using principal components analysis of household assets [22, 33]. A SFGR risk occupation was defined as one associated with livestock, dog, or dense vegetation contact, including butcher, farmer, gardener, milk supplier, rancher, wildlife warden, or veterinarian [4, 8]. We categorized continuous variables into quintiles and examined their association with both acute SFGR and SFGR exposure graphically. If a linear relationship was observed, continuous variables were used. If a clear nonlinear relationship was observed, quintile categorical variables were used. For visualization, we generated kernel density plots for age and acute SFGR.

We compared those with and without acute SFGR or SFGR exposure by univariate analysis using sociodemographic features, individual risk factor variables, and geospatial variables. We formulated a multivariable model using all variables examined in the univariate analysis and determined a final model for each outcome through backwards selection and comparison of the Akaike information criterion [34]. Subgroup analysis among those aged <2 years was carried out using the multivariable model.

### Data Management

Data were entered using the Cardiff Teleform system (Cardiff, Inc, Vista, California) into an Access database (Microsoft Corporation, Redmond, Washington). We evaluated each variable for improbable and inconsistent values. Statistical analysis was performed using Stata version 17.0 software (StataCorp, College Station, Texas).

### Research Ethics

Ethics approval was obtained from the Health Research Ethics Committee of the Kilimanjaro Christian Medical University College (protocol number 295), the Tanzanian National Health Research Ethics Committee of the National Institute for Medical Research (NIMR/HQ/R.8a/Vol.IX/1000), the Duke University Health System Institutional Review Board (Pro00016134), and the University of Otago Human Ethics Committee (Health) (H15/055).

## RESULTS

### Study Population, Testing, Demographics, and Clinical Data

**Figure 1.**
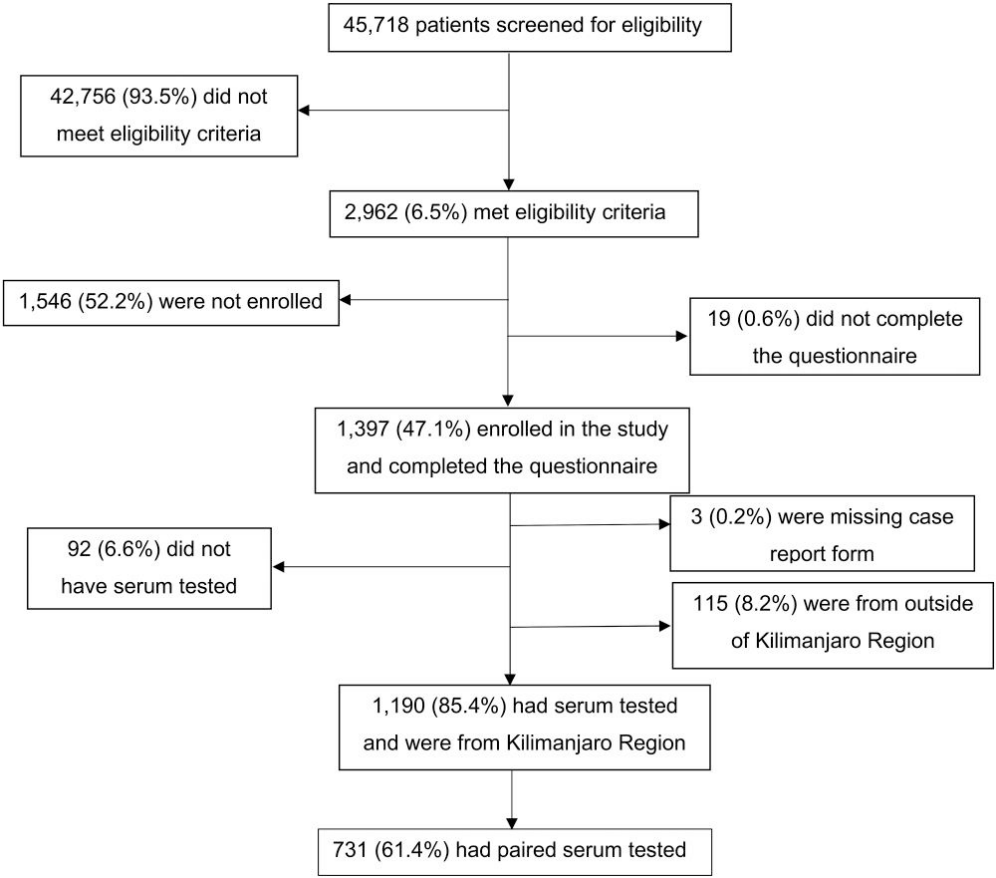
Study enrollment flow diagram, Kilimanjaro Region, Tanzania, 2012–2014.

**Figure 2.**
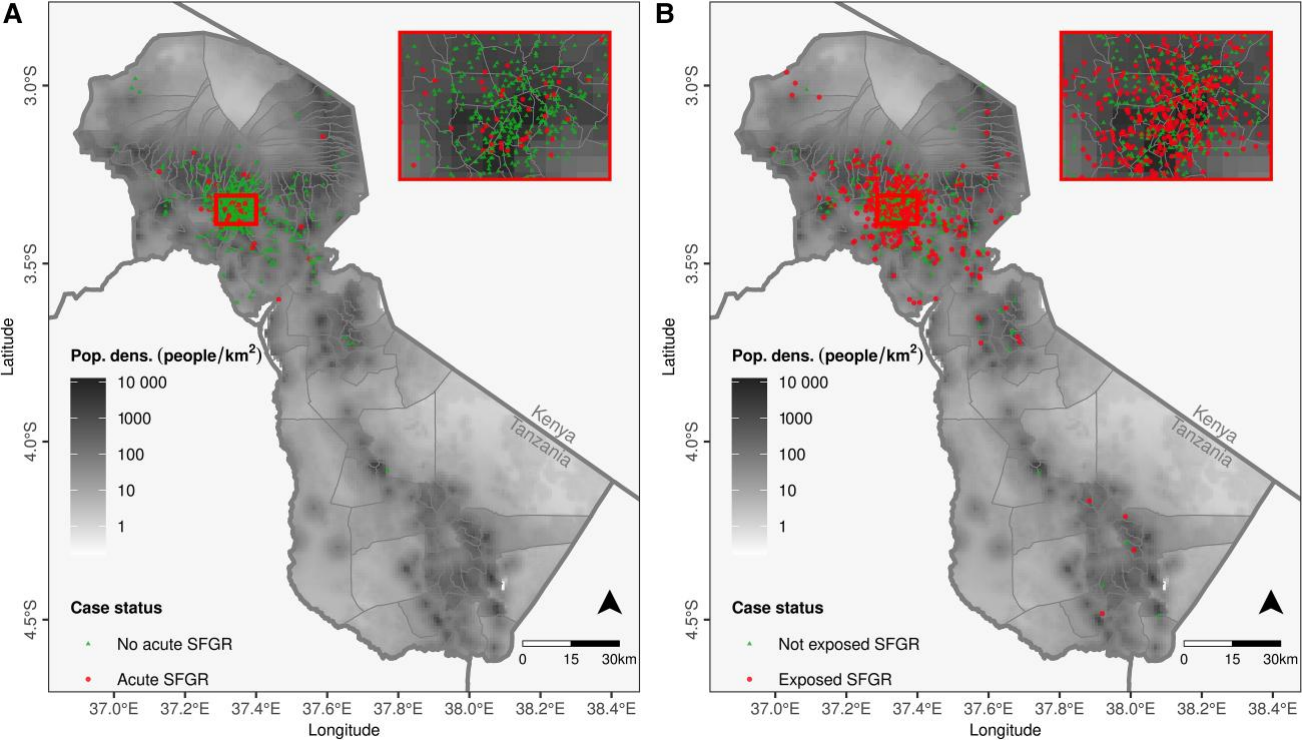
Location of participants with and without acute spotted fever group rickettsioses (SFGR) (*A*) and those with and without exposure to SFGR (*B*). Insets show the area including Moshi municipality district wards, Kilimanjaro Region, Tanzania, 2012–2014.

Of 30 413 outpatients and 15 305 inpatients screened, 2962 met eligibility criteria, and 1394 (47.1%) were enrolled and completed the risk factor questionnaire; among these, the final analysis included the 1190 (85.4%) who were residents of the Kilimanjaro Region and also had serum tested for SFGR by IFA (Figure 1). Of included participants, 646 (54.3%) were female, 618 (52.0%) were of the Chaga tribe, and 606 (50.9%) lived in Moshi municipality (Table 1). Of 731 participants with paired sera, 67 (9.2%) met the definition for acute SFGR while 650 of 1190 (54.6%) participants with at least a single serum sample met the definition for SFGR exposure. The locations of participants with and without acute SFGR and with and without exposure to SFGR are shown in Figure 2. Of 67 participants who with acute SFGR, 41 (61.2%) were inpatients, 22 (32.8%) had a fever for ≥7 days, 2 (5%) had lymphadenopathy, 5 (7.5%) had a rash, and 0 (0%) had an eschar.

**Table 1.** Comparison of Demographic and Clinical Characteristics Between Participants With and Without Paired Serum Immunofluorescent Antibody Testing for Spotted Fever Group Rickettsioses, Kilimanjaro Region, Tanzania, 2012–2014 (N =1190)

Variable	Paired (n = 731)		Not Paired (n = 459)		Overall (N = 1190)	
	no./No.	(%)	no./No.	(%)	no./No.	(%)
Demographics						
Age, y, median (range)	25.4	(0.2–93.5)	17.2	(0.2–100.2)	21.8	(0.3–100.2)
Female sex	398	(54.5)	248	(54.0)	646	(54.3)
Secondary education	131	(17.9)	59/458	(12.9)	190/1189	(16.0)
Tribe						
Chaga	391/730	(53.6)	227	(49.5)	618	(52.0)
Pare	100/730	(13.7)	75	(16.3)	175	(14.7)
Maasai	6/730	(0.8)	1	(0.2)	7	(0.6)
Sambaa	38/730	(5.2)	24	(5.2)	62	(5.2)
Other	195/730	(26.7)	132	(28.8)	327	(27.5)
Moshi municipality	359	(49.1)	247	(53.8)	606	(50.9)
Population density category						
Urban	429	(58.7)	271	(59.0)	700	(58.8)
Periurban	232	(31.7)	140	(30.5)	372	(31.3)
Rural	70	(9.6)	48	(10.5)	118	(9.9)
Socioeconomic status						
Lowest quintile	151/722	(20.9)	84/452	(18.6)	235/1174	(20.0)
2nd quintile	138/722	(19.1)	97/452	(21.5)	235/1174	(20.0)
3rd quintile	149/722	(20.6)	86/452	(19.0)	235/1174	(20.0)
4th quintile	147/722	(20.4)	88/452	(19.5)	235/1174	(20.0)
Highest quintile	137/722	(19.0)	97/452	(21.5)	234/1174	(19.9)
Clinical history						
Inpatient	433	(59.2)	270	(58.8)	703	(59.1)
Days of fever prior to presentation, median (range)	4	(0–120)	4	(0–90)	4	(0–120)
Fever duration ≥7 d	202	(27.6)	166	(36.2)	368	(30.9)
Rash present	47/729	(6.5)	40/458	(8.7)	87/1187	(7.3)
Lymphadenopathy	40/730	(5.5)	30	(6.5)	70/1189	(5.9)
Eschar	12	(1.6)	5/458	(1.1)	17/1189	(1.4)

Data are presented as No. (%) unless otherwise noted.

### Risk Factors for Acute SFGR

The univariate analysis of risk factors for acute SFGR is shown in Table 2. On multivariable analysis, the odds of acute SFGR were higher in the age group 0–2 years (adjusted odds ratios [aORs] for all 5 older age groups, <0.36; *P* < .011), in those with rural residence compared to urban residence (aOR, 4.1; *P* = .007), and those in areas with a maximum daily temperature <26°C (aORs for all higher temperature groups, <0.42; *P* < .035) (Table 3). The odds of acute SFGR were lower among participants who owned cattle compared to those who did not (aOR, 0.33; *P* = .050) and among those who lived in an area with a goat density of 7.9–36.1 head per km² (aOR, 0.34; *P* = .043). In subgroup analysis, of those aged <2 years (n = 24 acute SFGR, n = 73 no acute SFGR), no variables were statistically significantly associated with acute SFGR. Supplementary Figure 1 shows the kernel density plot for participants with and without acute SFGR <2 years of age.

**Table 2.** Univariate Logistic Regression of Potential Risk Factors for Acute Spotted Fever Group Rickettsioses Among Febrile Participants, Kilimanjaro Region, Tanzania 2012–2014 (n = 731)

Variable	Acute SFGR (n = 67)		No Acute SFGR (n = 664)		Univariate Analysis		
	no./No.	(%)	no./No.	(%)	OR	(95% CI)	*P* Value^a^
Demographics							
Age group, y							
<2	24	(35.8)	73	(11.0)	Ref	Ref	Ref
2–9	10	(14.9)	160	(24.1)	0.19	(.09–.42)	<.001
10–29	6	(9.0)	138	(20.8)	0.13	(.05–.34)	<.001
30–39	11	(16.4)	135	(20.3)	0.25	(.11–.53)	<.001
≥40	16	(23.9)	158	(23.8)	0.31	(.15–.61)	.001
Female sex	36	(53.7)	362	(54.5)	0.97	(.59–1.6)	.902
Secondary education	6	(9.0)	125	(18.8)	0.42	(.18–1.0)	.051
Chaga tribe	27	(40.3)	364	(54.8)	0.56	(.33–.93)	.025
Population density category							
Urban	41	(62.2)	388	(58.4)	Ref	Ref	Ref
Periurban	18	(26.9)	214	(32.2)	0.80	(.45–1.4)	.440
Rural	8	(11.9)	62	(9.3)	1.2	(.55–2.7)	.626
Socioeconomic status^b^							
Lowest quintile	10/66	(15.2)	138/656	(21.0)	Ref	Ref	Ref
2nd quintile	14/66	(21.2)	119/656	(18.1)	1.6	(.70–3.8)	.263
3rd quintile	16/66	(24.2)	144/656	(22.0)	1.5	(.67–3.5)	.309
4th quintile	11/66	(16.7)	133/656	(20.3)	1.1	(.47–2.8)	.771
Highest quintile	15/66	(22.7)	122/656	(18.6)	1.7	(.74–3.9)	.215
Potential SFGR exposure in the last month							
SFGR risk occupation^c^	10	(14.9)	145/663	(21.9)	0.63	(.31–1.3)	.189
Walking barefoot	25	(37.3)	307	(46.2)	0.69	(.41–1.2)	.164
Work in fields	9	(13.4)	167	(25.2)	0.46	(.22–.95)	.036
Work in garden	4	(6.0)	83	(12.5)	0.44	(.16–1.3)	.125
Dogs							
Own	8	(11.3)	124	(17.7)	0.59	(.28–1.3)	.175
Seen in village other than own	49	(74.2)	530	(79.8)	0.72	(.41–1.3)	.288
Cattle							
Own	9	(13.4)	154	(23.2)	0.51	(.25–1.1)	.072
Fed	5	(7.5)	71/663	(10.7)	0.67	(.26–1.7)	.410
Herd	0	(0)	10	(1.5)	…	…	
Seen in village other than own	36	(53.7)	447	(67.3)	0.56	(.34–.94)	.027
Goats							
Own	12	(17.9)	168	(25.3)	0.64	(.34–1.2)	.184
Fed	3	(4.5)	70/663	(10.6)	0.40	(.12–1.3)	.126
Herd	0	(0)	20	(3.0)	…	…	
Seen in village other than own	43	(64.2)	493	(74.4)	0.62	(.36–1.0)	.074
Sheep							
Own	3	(4.5)	50	(7.5)	0.58	(.17–1.9)	.364
Fed	1	(1.5)	18/663	(2.7)	0.54	(.07–4.1)	.555
Herd	0	(0)	3	(0.5)	…	…	
Seen in village other than own	22/66	(33.3)	223/662	(33.7)	0.98	(.58–1.7)	.954
Environmental variables							
Mean rainfall in last 3 mo, mm, median (range)	49.9	(5.2–225.7)	65.2	(6.3–309.8)	1.0	(1.0–1.0)	.926
Mean maximum temperature in last 3 mo^b^, °C							
14.6–<26.0	21	(31.3)	192	(19.4)	Ref	Ref	Ref
26.0–<27.6	9	(13.4)	138	(20.8)	0.40	(.18–.91)	.028
27.6–<29.6	13	(19.4)	137	(20.6)	0.58	(.28–1.2)	.149
29.6–<31.3	14	(20.9)	126	(19.0)	0.68	(.33–1.4)	.298
31.3–33.0	10	(14.9)	134	(20.2)	0.46	(.21–1.0)	.053
NDVI in prior 3 mo, median (range)	0.41	(0.21–0.80)	0.43	(0.18–0.84)	0.30	(.04–2.2)	.233
Cattle density, heads/km^2^, median (range)	556.9	(1.76–3140.9)	186.2	(0–4887.8)	1.0	(1.0–1.0)	.135
Goat density^b^, heads/km^2^							
0–<0.2	15	(22.4)	134	(20.2)	Ref	Ref	Ref
0.2–<7.9	9	(13.4)	148	(22.3)	0.54	(.23–1.3)	.164
7.9–<36.5	8	(11.9)	140	(21.1)	0.51	(.21–1.2)	.139
36.5–<1031.0	16	(23.9)	124	(18.7)	1.2	(.55–2.4)	.709
1031.0–2390.5	19	(28.4)	118	(17.8)	1.4	(.70–3.0)	.323
Sheep density^b^, heads/km^2^							
0–<0.3	15	(22.4)	136	(20.5)	Ref	Ref	Ref
0.3–<2.1	16	(23.9)	119	(18.0)	1.2	(.58–2.6)	.603
2.1–<3.7	15	(22.4)	135	(20.3)	1.0	(.47–2.1)	.985
3.7–<6.1	7	(10.5)	152	(22.9)	0.42	(.17–1.1)	.065
6.1–105.4	14	(20.9)	122	(18.4)	1.0	(.48–2.2)	.919
Elevation^b^, meters							
532–790	12	(17.9)	137	(20.6)	Ref	Ref	Ref
791–812	20	(20.9)	140	(21.1)	1.6	(.77–3.5)	.203
813–864	9	(13.4)	108	(16.3)	0.95	(.39–2.3)	.914
865–1001	17	(25.4)	137	(20.6)	1.4	(.65–3.1)	.379
1002–3375	9	(13.4)	142	(21.4)	0.72	(.30–1.8)	.479

Data are presented as No. (%) unless otherwise noted.

Abbreviations: CI, confidence interval; NDVI, normalized difference vegetation index; OR, odds ratio; SFGR, spotted fever group rickettsioses.

^a^Odds ratio and *P* value by univariate logistic regression.

^b^Nonlinear relationship observed so categorical quintile variables used.

^c^SFGR risk occupation was defined as reporting 1 of butcher, farmer, gardener, milk supplier, rancher, wildlife warden, or veterinarian as a primary or secondary occupation.

**Table 3.** Final Multivariable Logistic Regression Model of Potential Risk Factors for Acute Spotted Fever Group Rickettsioses Among Febrile Participants, Kilimanjaro Region, Tanzania 2012–2014 (n = 730)

Variable	Multivariable OR	(95% CI)	*P* Value
Age group, y			
<2	Ref	Ref	Ref
2–9	0.19	(.09–.45)	<.001
10–29	0.11	(.04–.30)	<.001
30–39	0.26	(.11–.61)	.002
≥40	0.36	(.16–.79)	.011
Chaga tribe	0.63	(.35–1.1)	.114
Population density category			
Urban	Ref	Ref	Ref
Periurban	1.7	(.80–3.7)	.163
Rural	4.1	(1.5–11.6)	.007
Work in fields	0.49	(.20–1.2)	.106
Cattle			
Own	0.33	(.11–1.0)	.050
Fed	3.0	(.73–12.5)	.126
Mean maximum temperature last 3 mo^a^, °C			
14.6–<26.0	Ref	Ref	Ref
26.0–<27.6	0.30	(.12–.71)	.006
27.6–<29.6	0.38	(.17–.85)	.019
29.6–<31.3	0.42	(.19–.94)	.035
31.3–33.0	0.31	(.13–.75)	.009
Goat density^a^, heads/km^2^			
0–<0.2	Ref	Ref	Ref
0.2–<7.9	0.44	(.16–1.2)	.110
7.9–<36.1	0.34	(.12–.97)	.043
36.1–<1031	0.93	(.41–2.1)	.865
1031.0–2390.5	1.8	(.80–3.8)	.160

Abbreviations: CI, confidence interval; OR, odds ratio.

^a^Nonlinear relationship observed so categorical quintile variables used.

### Risk Factors for SFGR Exposure

The univariate analysis of risk factors for acute SFGR is shown in Table 4. On multivariable analysis, odds of SFGR exposure were higher in those working in the garden (aOR, 1.8; *P* = .010), or seeing a dog in the village within the previous 30 days (aOR, 1.5; *P* = .010) (Table 5). Odds of SFGR exposure were lower in the age group 0–2 years (aORs for all 5 older age groups, >1.5; *P* < .026), female sex compared to male sex (aOR, 0.62; *P* < .001), or being from the Chaga tribe compared to other tribes (aOR, 0.68; *P* = .003). Living in an area with an annual mean NDVI of 0.4–<0.5 was associated with lower odds of SFGR exposure (aOR, 0.67; *P* = .041).

**Table 4.** Univariate Logistic Regression of Potential Risk Factors for Spotted Fever Group Rickettsioses Exposure Among Febrile Participants, Kilimanjaro Region, Tanzania 2012–2014 (N = 1190)

Variable	Exposed SFGR (n = 650)		Not Exposed SFGR (n = 540)		Univariate Analysis		
	No.	(%)	No.	(%)	OR	(95% CI)	*P* Value^a^
Demographics							
Age group, y							
<2	93	(14.3)	140	(25.9)	Ref	Ref	Ref
2–9	125	(19.2)	123	(22.8)	1.5	(1.1–2.2)	.021
10–29	141	(21.7)	94	(17.4)	2.3	(1.6–3.3)	<.001
30–39	132	(20.3)	76	(14.1)	2.6	(1.8–3.8)	<.001
≥40	159	(24.5)	107	(19.8)	2.2	(1.6–3.2)	<.001
Female sex	324	(49.9)	322	(59.6)	0.67	(.53–.85)	.001
Secondary education	111/649	(17.1)	79	(14.6)	1.2	(.88–1.6)	.247
Chaga tribe	311	(47.9)	307	(56.9)	0.70	(.55–.88)	.002
Population density category							
Urban	386	(59.4)	314	(58.2)	Ref	Ref	Ref
Periurban	200	(30.8)	172	(31.9)	0.95	(.73–1.2)	.666
Rural	64	(9.9)	54	(10.0)	0.96	(.65–1.4)	.855
Socioeconomic status^b^							
Lowest quintile	132/638	(20.7)	103/536	(19.2)	Ref	Ref	Ref
2nd quintile	126/638	(19.8)	109/536	(20.3)	0.90	(.63–1.3)	.578
3rd quintile	123/638	(19.4)	111/536	(20.7)	0.87	(.61–1.3)	.459
4th quintile	138/638	(21.6)	97/536	(18.1)	1.1	(.77–1.6)	.576
Highest quintile	118/638	(18.5)	116/536	(21.6)	0.79	(.55–1.1)	.213
Potential SFGR exposure in the last month							
SFGR risk occupation^c^	143	(22.0)	89	(16.5)	1.4	(1.1–1.9)	.016
Walking barefoot	273	(42.0)	240	(44.4)	0.91	(.72–1.1)	.397
Work in fields	155	(23.9)	96	(17.8)	1.4	(1.1–1.9)	.011
Work in garden	82	(12.6)	36	(6.7)	2.0	(1.3–3.0)	.001
Dogs							
Own	99	(15.2)	89	(16.5)	0.91	(.67–1.2)	.556
Seen in village other than own	527	(81.3)	396	(73.3)	1.6	(1.2–2.1)	.001
Cattle							
Own	140	(21.5)	112	(20.7)	1.0	(.79–1.4)	.737
Fed	70	(10.8)	40/539	(7.4)	1.5	(1.0–2.3)	.048
Herd	9/649	(1.4)	4	(0.7)	1.9	(.58–6.2)	.294
Seen in village other than own	427/648	(65.9)	331/539	(61.4)	1.2	(.96–1.5)	.109
Goats							
Own	146	(22.5)	132	(24.4)	0.90	(.68–1.2)	.421
Fed	59	(9.1)	43	(8.0)	1.2	(.76–1.7)	.501
Herd	16	(2.5)	11	(2.0)	1.2	(.56–2.6)	.622
Seen in village other than own	473/647	(73.1)	375/539	(69.6)	1.2	(.92–1.5)	.180
Sheep							
Own	53	(8.2)	41	(7.6)	1.1	(.71–1.7)	.721
Fed	23	(3.5)	10/539	(1.9)	1.9	(.92–4.1)	.084
Herd	4/648	(0.6)	4	(0.7)	0.83	(.21–3.3)	.796
Seen in village other than own	231/647	(35.7)	164/537	(30.5)	1.3	(.99–1.6)	.061
Environmental variables							
Annual mean rainfall^b^, mm							
27.5–<51.0	135	(20.8)	103	(19.1)	Ref	Ref	Ref
51.0–<54.6	151	(23.2)	104	(19.3)	1.1	(.77–1.6)	.575
54.6–<65.7	169	(26.0)	162	(30.0)	0.80	(.57–1.1)	.182
65.7–<78.8	76	(11.7)	71	(13.2)	0.82	(.54–1.2)	.336
78.8–134.0	119	(18.3)	100	(18.5)	0.91	(.63–1.3)	.608
Annual mean maximum temperature^b^, °C							
14.4–<27.5	131	(20.2)	107	(19.8)	Ref	Ref	Ref
27.5–<29.1	136	(20.9)	119	(22.0)	0.93	(.65–1.3)	.704
29.1–<29.3	154	(23.7)	130	(24.1)	0.97	(.68–1.4)	.852
29.3–<29.4	91	(14.0)	86	(15.9)	0.86	(.59–1.3)	.464
29.4–30.1	138	(21.2)	98	(18.1)	1.2	(.80–1.7)	.451
Annual mean NDVI^b^							
0.2–<0.3	140	(21.5)	98	(18.2)	Ref	Ref	Ref
0.3–<0.4	140	(21.5)	98	(18.2)	1.0	(.69–1.4)	1.000
0.4–<0.5	115	(17.7)	123	(22.8)	0.65	(.46–.94)	.022
0.5–<0.6	131	(20.2)	107	(19.8)	0.86	(.60–1.2)	.405
0.6–0.8	124	(19.1)	114	(21.1)	0.76	(.53–1.1)	.140
Cattle density, heads/km^2^, median (range)	221.6	(0–4887.8)	237.9	(0–4887.8)	1.0	(1.0–1.0)	.548
Goat density, heads/km^2^, median (range)	18.8	(0–2390.5)	17.0	(0–2390.5)	1.0	(1.0–1.0)	.189
Sheep density^b^, heads/km^2^							
0–<0.2	123	(19.4)	112	(20.7)	Ref	Ref	Ref
0.2–<2.1	128	(19.7)	113	(20.9)	1.0	(.70–1.4)	.970
2.1–<3.7	141	(21.7)	94	(17.4)	1.3	(.93–1.9)	.122
3.7–<6.1	126	(19.4)	115	(21.3)	0.97	(.68–1.4)	.885
6.1–105.4	129	(19.9)	106	(19.6)	1.1	(.75–1.6)	.670
Elevation, meters, median (range)	827.5	(688–2148)	831.5	(532–3375)	1.0	(1.0–1.0)	.315

Data are presented as No. (%) unless otherwise noted.

Abbreviations: CI, confidence interval; NDVI, normalized difference vegetation index; OR, odds ratio; SFGR, spotted fever group rickettioses.

^a^Odds ratio and *P* value by univariate logistic regression.

^b^Nonlinear relationship observed so categorical quintile variables used.

^c^SFGR risk occupation was defined as reporting 1 of butcher, farmer, gardener, milk supplier, rancher, wildlife warden, or veterinarian as a primary or secondary occupation.

**Table 5.** Final Multivariable Logistic Regression Model of Potential Risk Factors for Spotted Fever Group Rickettsioses Exposure Among Febrile Participants, Kilimanjaro Region, Tanzania 2012–2014 (n = 1188)

Variable	Multivariable OR	(95% CI)	*P* Value
Age group, y			
<2	Ref	Ref	Ref
2–9	1.5	(1.1–2.2)	.026
10–29	2.2	(1.5–3.3)	<.001
30–39	2.2	(1.9–4.2)	<.001
≥40	2.3	(1.6–3.4)	<.001
Female sex	0.62	(.48–.79)	<.001
Chaga tribe	0.68	(.53–.88)	.003
Work in garden	1.8	(1.1–2.7)	.010
Dogs seen in village other than own	1.5	(1.1–2.0)	.010
Annual mean NDVI^a^			
0.2–<0.3	Ref	Ref	Ref
0.3–<0.4	1.1	(.74–1.6)	.688
0.4–<0.5	0.67	(.46–.98)	.041
0.5–<0.6	0.89	(.61–1.3)	.565
0.6–0.8	0.75	(.50–1.1)	.164

Abbreviations: CI, confidence interval; NDVI, normalized difference vegetation index; OR, odds ratio.

^a^Nonlinear relationship observed so categorical quintile variables used.

## DISCUSSION

Consistent with past research, we confirmed that SFGR is a common cause of severe febrile illness in Kilimanjaro, Tanzania [1, 2]. In our study, 9.1% met the definition for acute SFGR and 54.6% met the definition for SFGR exposure. For acute SFGR, age <2 years, residing rurally, and living in an area with a lower maximum daily temperature were each identified as important risk factors for disease. Age ≥2 years was associated with SFGR exposure, as was working in the garden and seeing dogs in the village. Female sex and being from the Chaga tribe were identified as being protective for SFGR exposure.

Of considerable importance for prevention and clinical management, we found that acute SFGR infections were concentrated among infants and young children. While we found that the median age of those with acute SFGR was higher than those without acute SFGR in an earlier study in Moshi, Tanzania [2], the findings of our present study are consistent with research from Kenya demonstrating that acute SFGR accounted for 22.4% of undifferentiated fever among participants aged 1–12 years [35].

As might be expected in a population with acute disease early in life, the odds of SFGR exposure increased with age. That SFGR exposure increases with age is consistent with a study from the Mbeya Region of southwestern Tanzania [9]. Taken together, the findings suggest that infections early in life may provide protection against more severe manifestations of acute SFGR subsequently, perhaps boosted by regular reexposure. While it is unknown how long primary infection protects an individual from acute illness, nor how long IgG remains detectable after exposure [36], in Rocky Mountain spotted fever, IgG titers have been detected >1 year after an acute infection [37]. Overall, our findings suggest that greater attention should be paid to SFGR as a cause of fever among infants and young children in Tanzania and to identifying risk factors for disease particular to this age group.

Acute SFGR was associated with residing rurally and SFGR exposure with working in a garden. Previously, in rural central Africa, SFGR exposure has been associated with working in agriculture [8]. In addition, *A variegatum* vectors of *R africae* are found in greater abundance in cropland in Tanzania compared to other areas of land use [38]. Our findings are consistent with risk for disease and exposure being linked to *R africae* tick vector habitats.

Both *R conorii* and *R africae* are known to be present in Tanzania [10, 39] and there may be as yet undiscovered SFGR species in Tanzania. Which of these species predominates as a cause of SFGR is presently unknown. In our multivariable analysis, seeing dogs in the village was associated with SFGR exposure. Previously in Europe, where *R conorii* dominates, a higher seroprevalence of SFGR has been observed in urban areas compared with rural areas, thought to be associated with high urban dog density [39]. In our study, dog ownership was not associated with SFGR. We speculate that either dog ownership is a poor marker of dog exposure or that, in our study, seeing dogs in the village was perhaps reflecting a participant’s mobility rather than dog exposure itself. In Tanzania, dog ownership has also previously been linked with cattle ownership [40]. Previous research demonstrating higher cattle density associated with higher prevalence of SFGR exposure suggested that *R africae* may be the dominant agent of SFGR in southern Tanzania [9]. However, we found no association between cattle exposures and increased odds of SFGR. Indeed cattle ownership, a poor marker of cattle exposure, was associated with lower odds of acute SFGR in our study. Further research is needed to characterize the agents of SFGR in Tanzania and to disentangle potentially overlapping exposures.

While female sex was protective for SFGR exposure in our study, sex was not associated with acute SFGR. The protective effect of female sex against SFGR exposure has been noted previously in both Tanzania and Namibia [7, 9].

Being from the Chaga tribe compared to other tribes was found to be protective against SFGR exposure. An analysis of livestock production systems in northern Tanzania showed that the cluster that contained Chaga-led households had smaller cattle herd sizes, lived in areas with a higher cattle density, and had more local cropland cover compared to the mean of other clusters [21]. In addition, the Chaga cluster experienced less hunger and had a higher ownership of household latrines than other clusters, all likely surrogates for wealth [21]. In our analysis, cattle density was not a risk factor for acute SFGR or SFGR exposure and the socioeconomic economic score had no association with either acute SFGR or SFGR exposure. The relationship between tribe and SFGR risk is clearly complex. Further research is needed to elucidate the drivers of lower odds of SFGR exposure among members of the Chaga tribe.

In our study, maximum daily temperatures <26°C in the 3 months prior to enrollment were associated with acute SFGR. By contrast, elsewhere in Tanzania, increasing ambient air temperature was a risk factor for SFGR exposure [9], likely mediated by increased tick affinity to hosts [17, 18]. However, the relationship between temperature and tick presence appears to be complex. *A*  *variegatum* ticks are generally found where the minimum monthly temperature remains above 10°C year-round [19]. In Zambia, *A variegatum* larval mortality is particularly high during the hot months when soil temperatures reach 30°C [16]. Tick nymphae exhibit more questing and active host-seeking during the cooler early mornings and late afternoons compared to the warmer middle of the day [16]. Our finding of acute SFGR risk at maximum daily temperatures <26°C may suggests that higher temperatures are not favorable for tick survival, host-seeking, or questing.

Our study had a number of limitations. A large proportion (35.8%) of the acute SFGR occurred in participants aged <2 years and our questionnaire was not designed to capture developmental milestone-related exposures in this age group. The questionnaire was designed to address exposures for a range of bacterial zoonoses, so was not optimized for all relevant SFGR-associated exposures. Future studies should focus on a comprehensive range of SFGR exposures, including those relevant to infants and young children, and be powered to detect effects in this age group. Since we enrolled participants seeking healthcare at 2 referral hospitals, our participants may not be representative of the general population of the Kilimanjaro Region. Persons with SFGR living in and around Moshi may have been more likely to seek care at study hospitals than those living farther away, as has been demonstrated in Kenya [41]. In the Kilimanjaro Region, febrile participants from the highest wealth quintile were more likely to seek hospital care compared with less wealthy persons [42]. Rural persons were also not well represented among study participants. We relied on surrogates of tick exposure, such as measures of exposure to tick hosts, since directly measuring exposure to ticks is difficult. Not all participants had paired serum available for testing, so undiagnosed acute SFGR may have contaminated the comparison group for the SFGR exposure analysis. While demographic characteristics were similar between those that had paired serum and those that did not, there were differences in some domains (Table 1).

SFGR is common in the Kilimanjaro Region, and infants and young children were found to have a particularly high risk for acute disease. Rural residence was associated with acute SFGR and working in the garden with SFGR exposure. Female sex was protective for SFGR exposure. A high index of suspicion should be maintained for acute SFGR among febrile infants and young children in this setting. There should be a low threshold for SFGR-targeted diagnostics and a treatment of febrile infants and young children in the Kilimanjaro Region. Prevention measures need to be co-designed in light of local conditions, including assessing community acceptability through social science research. These measures may include the use of insect repellents on both people and impregnated into clothes; identification of tick attachment and early tick removal, including by parents of infants and young children; use of acaricides in domestic animals and livestock; and attempts to keeping the skin covered when entering tick habitats [36, 43]. Future research in Tanzania should focus on exposures early in life, particularly specific to prevention in infants and young children, and on identifying the rickettsial species that is the dominant cause of SFGR, so that interventions can be tailored to the specific transmission pathways.

## Supplementary Material

ofae664_Supplementary_Data

## Supplementary Data

Supplementary materials are available at *Open Forum Infectious Diseases* online. Consisting of data provided by the authors to benefit the reader, the posted materials are not copyedited and are the sole responsibility of the authors, so questions or comments should be addressed to the corresponding author.

## Notes

***Acknowledgments.*** The authors would like to thank those involved in recruitment, laboratory work, data management, and study administration, including Godfrey S. Mushi, Flora W. Mboya, Lilian E. Ngowi, Winfrida H. Shirima, Michael E. Butoyi, Anna H. Mwalla, Miriam L. Barabara, Ephrasia Mariki, Tumsifu G. Tarimo, Yusuf S. Msuya, Leila J. Sawe, Aaron E. Tesha, Luig J. Mbuya, Edward M. Singo, Isaac A. Afwamba, Thomas M. Walongo, Remigi P. Swai, Augustine M. Musyoka, Philoteus A. Sakasaka, O. Michael Omondi, Enoch J. Kessy, Alphonse S. Mushi, Robert S. Chuwa, Cynthia A. Asiyo, Frank M. Kimaro, and Francis P. Karia. In addition, we would like to thank the study participants as well as the clinical staff and administration at Kilimanjaro Christian Medical Centre and Mawenzi Regional Referral Hospital for their support during this study.

***Prior presentation.*** 73rd American Society of Tropical Medicine and Hygiene annual meeting, Abstract 7605, New Orleans, Louisiana, 13–17 November 2024.

***Data availability.*** All data contributing to statistical analyses are available from http://dx.doi.org/10.5525/gla.researchdata.1779. Patient location data used for figures in the paper are not shared to protect participant anonymity. For any further questions, contact the corresponding author.

***Disclaimer.*** The findings and conclusions in this report are those of the authors and do not necessarily represent the official position of the US Centers for Disease Control and Prevention (CDC). Use of trade names and commercial sources is for identification only and does not imply endorsement by the US Department of Health and Human Services or the CDC. The funders had no role in study design, data collection and analysis, decision to publish, or preparation of the manuscript.

***Financial support*.** This work was supported by the joint US National Institutes of Health–National Science Foundation Ecology and Evolution of Infectious Diseases program (grant number R01 TW009237) and the Research Councils UK, Department for International Development (UK), and UK Biotechnology and Biological Sciences Research Council (grant numbers BB/J010367/1, BB/L018926, BB/L017679, and BB/L018845). This work was also supported in part by a National Institutes of Health International Studies on AIDS Associated Co-infections award (grant number U01 AI062563) and by the Bill & Melinda Gates Foundation–funded Typhoid Fever Surveillance in Sub-Saharan Africa Program (grant number OPPGH5231). T. R. B. received support through a University of Otago Frances Cotter Scholarship and the University of Otago Postgraduate Publishing Bursary (Master's). M. P. R. received support from the National Institutes of Health (research training grant numbers R25 TW009337 and R25 TW009343) funded by the Fogarty International Center and from the National Institute of Allergy and Infectious Diseases (award number K23116869). M. P. R. and J. A. C. received support from the National Institute for Allergy and Infectious Diseases (grant number R01 AI121378).

## References

[1] Crump JA, Morrissey AB, Nicholson WL, et al Etiology of severe non-malaria febrile illness in northern Tanzania: a prospective cohort study. PLoS Negl Trop Dis 2013; 7:e2324. 23875053 10.1371/journal.pntd.0002324 PMC3715424

[2] Prabhu M, Nicholson WL, Roche AJ, et al Q fever, spotted fever group, and typhus group rickettsioses among hospitalized febrile patients in northern Tanzania. Clin Infect Dis 2011; 53:e8–15. 21810740 10.1093/cid/cir411 PMC3148261

[3] Pisharody S, Rubach MP, Carugati M, et al Incidence estimates of acute Q fever and spotted fever group rickettsioses, Kilimanjaro, Tanzania, from 2007 to 2008 and from 2012 to 2014. Am J Trop Med Hyg 2021; 106:494–503. 34929672 10.4269/ajtmh.20-1036 PMC8832940

[4] Jensenius M, Fournier PE, Vene S, et al African tick bite fever in travelers to rural sub-Equatorial Africa. Clin Infect Dis 2003; 36:1411–7. 12766836 10.1086/375083

[5] Jensenius M, Fournier PE, Kelly P, Myrvang B, Raoult D. African tick bite fever. Lancet Infect Dis 2003; 3:557–64. 12954562 10.1016/s1473-3099(03)00739-4

[6] Jensenius M, Hoel T, Raoult D, et al Seroepidemiology of *Rickettsia africae* infection in Norwegian travellers to rural Africa. Scand J Infect Dis 2002; 34:93–6. 11928860 10.1080/00365540110077029

[7] Noden BH, Tshavuka FI, Van Der Colf BE, Chipare I, Wilkinson R. Exposure and risk factors to *Coxiella burnetii*, spotted fever group and typhus group rickettsiae, and *Bartonella henselae* among volunteer blood donors in Namibia. PLoS One 2014; 9:e108674. 25259959 10.1371/journal.pone.0108674 PMC4178180

[8] Ndip LM, Biswas HH, Nfonsam LE, et al Risk factors for African tick-bite fever in rural Central Africa. Am J Trop Med Hyg 2011; 84:608–13. 21460018 10.4269/ajtmh.2011.10-0191 PMC3062457

[9] Heinrich N, Dill T, Dobler G, et al High seroprevalence for spotted fever group rickettsiae, is associated with higher temperatures and rural environment in Mbeya region, southwestern Tanzania. PLoS Negl Trop Dis 2015; 9:e0003626. 25849718 10.1371/journal.pntd.0003626 PMC4388512

[10] Parola P, Paddock CD, Socolovschi C, et al Update on tick-borne rickettsioses around the world: a geographic approach. Clin Microbiol Rev 2013; 26:657–702. 24092850 10.1128/CMR.00032-13 PMC3811236

[11] Rolfe RJ, Sheldon SW, Kingry LC, et al Metagenomic detection of bacterial zoonotic pathogens among febrile patients, Tanzania, 2007–2009. Emerg Infect Dis 2024; 30:1599–608. 39043406 10.3201/eid3008.240529 PMC11286057

[12] Spernovasilis N, Markaki I, Papadakis M, Mazonakis N, Ierodiakonou D. Mediterranean spotted fever: current knowledge and recent advances. Trop Med Infect Dis 2021; 6:172. 34698275 10.3390/tropicalmed6040172 PMC8544691

[13] Maina AN, Jiang J, Omulo SA, et al High prevalence of *Rickettsia africae* variants in *Amblyomma variegatum* ticks from domestic mammals in rural western Kenya: implications for human health. Vector Borne Zoonotic Dis 2014; 14:693–702. 25325312 10.1089/vbz.2014.1578 PMC4208559

[14] Berrian AM, Martínez-López B, Quan V, et al Risk factors for bacterial zoonotic pathogens in acutely febrile patients in Mpumalanga province, South Africa. Zoonoses Public Health 2019; 66:458–69. 30859717 10.1111/zph.12577

[15] Moorthy GS, Rubach MP, Maze MJ, et al Prevalence and risk factors for Q fever, spotted fever group rickettsioses, and typhus group rickettsioses in a pastoralist community of northern Tanzania, 2016–2017. Trop Med Int Health 2024; 29:365–76. 38480005 10.1111/tmi.13980 PMC11073910

[16] Pegram RG, Banda DS. Ecology and phenology of cattle ticks in Zambia: development and survival of free-living stages. Exp Appl Acarol 1990; 8:291–301. 2350995 10.1007/BF01202139

[17] Backus LH, López Pérez AM, Foley JE. Effect of temperature on host preference in two lineages of the brown dog tick, *Rhipicephalus sanguineus*. Am J Trop Med Hyg 2021; 104:2305–11. 33819179 10.4269/ajtmh.20-1376 PMC8176482

[18] Socolovschi C, Raoult D, Parola P. Influence of temperature on the attachment of *Rhipicephalus sanguineus* ticks on rabbits. Clin Microbiol Infect 2009; 15:326–7. 19438617 10.1111/j.1469-0691.2008.02260.x

[19] Rahajarison P, Arimanana AH, Raliniaina M, Stachurski F. Survival and moulting of *Amblyomma variegatum* nymphs under cold conditions of the Malagasy highlands. Infect Genet Evol 2014; 28:666–75. 24999236 10.1016/j.meegid.2014.06.022

[20] National Bureau of Statistics of Tanzania . Kilimanjaro region: basic demographic and socio-economic profile: 2012 population and housing census. Dar es Salaam, Tanzania: National Bureau of Statistics; 2016.

[21] de Glanville WA, Davis A, Allan KJ, et al Classification and characterisation of livestock production systems in northern Tanzania. PLoS One 2020; 15:e0229478. 33378382 10.1371/journal.pone.0229478 PMC7773236

[22] Cash-Goldwasser S, Maze MJ, Rubach MP, et al Risk factors for human brucellosis in northern Tanzania. Am J Trop Med Hyg 2018; 98:598–606. 29231152 10.4269/ajtmh.17-0125 PMC5929176

[23] Maze MJ, Cash-Goldwasser S, Rubach MP, et al Risk factors for human acute leptospirosis in northern Tanzania. PLoS Negl Trop Dis 2018; 12:e0006372. 29879114 10.1371/journal.pntd.0006372 PMC5991637

[24] Paris DH, Dumler JS. State of the art of diagnosis of rickettsial diseases: the use of blood specimens for diagnosis of scrub typhus, spotted fever group rickettsiosis, and murine typhus. Curr Opin Infect Dis 2016; 29:433–9. 27429138 10.1097/QCO.0000000000000298 PMC5029442

[25] Centers for Disease Control and Prevention . Spotted fever rickettsiosis (including Rocky Mountain spotted fever) 2020 case definition. 2020. Available at: https://ndc.services.cdc.gov/case-definitions/spotted-fever-rickettsiosis-2020/. Accessed 19 October 2022.

[26] WorldPop . Global High Resolution Population Denominators Project. 2018. Available at: 10.5258/SOTON/WP00674. Accessed 7 November 2023.

[27] Moraga P, Cano J, Baggaley RF, et al Modelling the distribution and transmission intensity of lymphatic filariasis in sub-Saharan Africa prior to scaling up interventions: integrated use of geostatistical and mathematical modelling. Parasit Vectors 2015; 8:560. 26496983 10.1186/s13071-015-1166-x PMC4620019

[28] Fick SE, Hijmans RJ. WorldClim 2: new 1-km spatial resolution climate surfaces for global land areas. Int J Climatol 2017; 37:4302–15.

[29] Didan K. MOD13Q1 MODIS/Terra Vegetation Indices 16-Day L3 Global 250 m SIN Grid V006. 2015. Available at: 10.5067/MODIS/MOD13Q1.006. Accessed 11 July 2023.

[30] Farr TG, Rosen PA, Caro E, et al The Shuttle Radar Topography Mission. Rev Geophys 2007; 45, RG2004.

[31] Robinson TP, Wint GRW, Conchedda G, et al Mapping the global distribution of livestock. PLoS One 2014; 9:e96084. 24875496 10.1371/journal.pone.0096084 PMC4038494

[32] Textor J, van der Zander B, Gilthorpe MS, Liśkiewicz M, Ellison GT. Robust causal inference using directed acyclic graphs: the R package ‘dagitty’. Int J Epidemiol 2016; 45:1887–94. 28089956 10.1093/ije/dyw341

[33] Vyas S, Kumaranayake L. Constructing socio-economic status indices: how to use principal components analysis. Health Policy Plan 2006; 21:459–68. 17030551 10.1093/heapol/czl029

[34] Burnham KP, Anderson DR. Information and likelihood theory: a basis for model selection and inference. In: Model selection and multimodel inference. New York: Springer; 2002:49–97.

[35] Maina AN, Farris CM, Odhiambo A, et al Q fever, scrub typhus, and rickettsial diseases in children, Kenya, 2011–2012. Emerg Infect Dis 2016; 22:883–6. 27088502 10.3201/eid2205.150953 PMC4861507

[36] Biggs HM, Behravesh CB, Bradley KK, et al Diagnosis and management of tickborne rickettsial diseases: Rocky Mountain spotted fever and other spotted fever group rickettsioses, ehrlichioses, and anaplasmosis—United States. MMWR Rec Rep 2016; 65:1–44. 10.15585/mmwr.rr6502a1 27172113

[37] Clements ML, Dumler JS, Fiset P, Wisseman CL, Snyder MJ, Levine MM. Serodiagnosis of Rocky Mountain spotted fever: comparison of IgM and IgG enzyme-linked immunosorbent assays and indirect fluorescent antibody test. J Infect Dis 1983; 148:876–80. 6415180 10.1093/infdis/148.5.876

[38] Lynen G, Zeman P, Bakuname C, et al Cattle ticks of the genera *Rhipicephalus* and *Amblyomma* of economic importance in Tanzania: distribution assessed with GIS based on an extensive field survey. Exp Appl Acarol 2007; 43:303–19. 18044004 10.1007/s10493-007-9123-9

[39] Raoult D, Toga B, Chaudet H, Chiche-Portiche C. Rickettsial antibody in southern France: antibodies to *Rickettsia conorii* and *Coxiella burnetii* among urban, suburban and semi-rural blood donors. Trans R Soc Trop Med Hyg 1987; 81:80–1. 3445329 10.1016/0035-9203(87)90290-2

[40] Sikana L, Lembo T, Hampson K, et al Dog ownership practices and responsibilities for children's health in terms of rabies control and prevention in rural communities in Tanzania. PLoS Negl Trop Dis 2021; 15:e0009220. 33690720 10.1371/journal.pntd.0009220 PMC7946275

[41] Bigogo G, Audi A, Aura B, Aol G, Breiman RF, Feikin DR. Health-seeking patterns among participants of population-based morbidity surveillance in rural western Kenya: implications for calculating disease rates. Int J Infect Dis 2010; 14:e967–73. 20800525 10.1016/j.ijid.2010.05.016

[42] Madut DB, Rubach MP, Hertz JT, et al Healthcare utilization for febrile syndromes in northern Tanzania: a randomized multistage population-based cluster survey [Abstract LB-5250]. In: 71st American Society of Tropical Medicine and Hygiene Annual Meeting, Seattle, WA, 30 October–3 November 2022.

[43] Centers for Disease Control and Prevention . About other spotted fever rickettsioses. 2020. Available at: https://www.cdc.gov/other-spotted-fever/about/index.html?CDC_AAref_Val=https://www.cdc.gov/otherspottedfever/prevention/index.html. Accessed 14 December 2022.

