# Supplementary material for: Risk Factors for Spotted Fever Group Rickettsioses in Kilimanjaro Region, Tanzania
Source: Open Forum Infect Dis. 2024 Nov 13;11(12):ofae664. doi: 10.1093/ofid/ofae664 (PMC11651152; doi:10.1093/ofid/ofae664)
Supplement: ofae664_Supplementary_Data [file ofae664_supplementary_data.zip › ofae664_Supplementary_Data.docx]

Supplementary Figure 1 Kernel density plots by age for febrile participants aged <2 years with and without acute spotted fever group rickettsioses, Kilimanjaro Region, Tanzania, 2012-2014 (N=97)


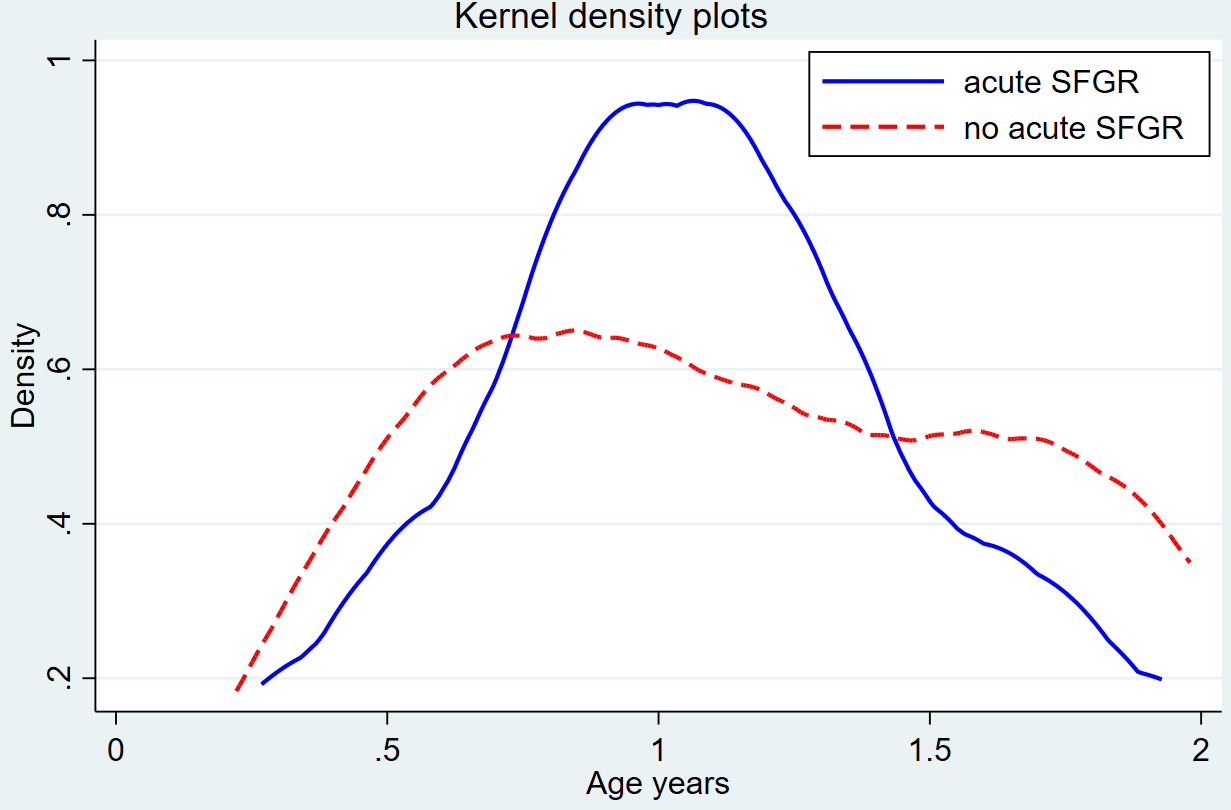
**Notes:** SFGR: spotted fever group rickettsioses
